# Barriers of Critical Thinking in Medical Students' Curriculum from the Viewpoint of Medical Education Experts: A Qualitative Study

**DOI:** 10.30476/jamp.2020.83053.1080

**Published:** 2020-04

**Authors:** AFSHINEH KASALAEI, MITRA AMINI, PARISA NABEIEI, LEILA BAZRAFKAN, HOURI MOUSAVINEZHAD

**Affiliations:** 1 Shiraz University of Medical Sciences, Shiraz, Iran; 2 Clinical Education Research Center, Shiraz University of Medical Sciences, Shiraz, Iran; 3 Cardiovascular Research Centre, Shiraz University of Medical Sciences, Shiraz, Iran

**Keywords:** Barriers, Thinking, Critical thinking, Curriculum, Medical education

## Abstract

**Introduction:**

The widespread developments of the twenty-first century have been accompanied by the presentation of intellectual patterns and theories and new achievements. These new achievements emphasize the skill of thinking at high levels, especially in the educational system of universities. This skill is essential for medical students; therefore, the present study aimed to investigate the qualitative barriers of critical thinking in medical students' curriculum.

**Methods:**

This is a qualitative study in which the content analysis method has been used. Participants of this study included 11 medical education experts and medical students (6 females and 5 males) who were selected through a semi-structured interview and purposeful sampling. The data analysis method was conventional content analysis. In the next part, by more investigation of the data, various obtained concepts will be presented in the form of themes, categories, and subcategories.

**Results:**

We obtained two themes (socio-cultural conditions and traditional and unchanging system of education), eight categories and 14 subcategories. Also, these categories were resistance to critical society, intellectual tension, personality characteristics, lack of understanding of society's need for criticism, the rule of traditional teaching pattern, lack of critical thinking skills, ineffective evaluation, and difficulty of critical thinking training.

**Conclusion:**

Given the results and the main emphasis of curriculum planners on incorporating high-level critical thinking and revision skills into the curriculum, the country's academic education system requires a change in the thinking style, research, deepening critical thinking, and a change in teachers' attitudes toward curriculum designing (goals, content, teaching and evaluation methods); also, it is suggested that the authorities should pay attention to the need to develop and utilize critical thinking skills in the learners’ education.

## Introduction

The widespread and vast developments of the 21st century are accompanied by the presentation of intellectual patterns and theories and the production of modern science and technology. One of the new achievements of this century is emphasis on thinking methods, especially in the educational system ( 1 ).

Critical education is a relatively modern theory developed by educators such as Paulo Freiere, Henry Giroux, Peter McLaren, Michael Apple, and Douglass Kellner based on the principles of critical theory ( 2 ). In this type of education, wisdom, critique, and interpretation are considered as valuable educational goals. The primary approach of this tutorial is, as mentioned before, toward critical thinking. In this type of training, the use of memory and old knowledge is diminished, and learners can analyze, evaluate, and interpret the material. Accordingly, the training of the criticizer and wise students is the first goal of university education to confront the changing society in this age of multiple information explosion ( 3 ).

Critical thinking is a type of high-level thinking skills used by students, i.e. they use personal perspectives and approaches rather than simple acceptance without evaluating the others' judgments, attitudes, and information ( 4 ). The central core of critical thinking is cognitive skills such as interpretation, analysis, evaluation, inference, explanation, and self-regulation.

Critical thinking in the field of medical sciences is a kind of cognitive activity for understanding and evaluating the phenomena based on reasoning and analysing ( 5 - 6 ). Having a critical thinking skill for a physician helps him make the right clinical decision and provide the best care in the patient care process ( 7 - 8 ). The ability to solve a problem at the patient's bedside is valuable in the health process.

Designing the curricula can reflect the important belief in medical students that they should learn these experiences not only to maximize their potential, but also use it as a general skill in classrooms, and conduct this vital issue to the other areas, their other life aspects, and future career ( 9 , 10 ). With this in mind, the importance of the students' curriculum in fostering the students' critical thinking becomes apparent.

Therefore, the institution of education in academic education must carry out its mission to review the goals, content and educational materials, methods of teaching-learning, and the evaluation system and everything related to the curriculum. It should be noticed that the surface change will not be responsive to the revision of the curriculum and that fundamental and logical changes in all curriculum processes are essential ( 11 , 12 ).

The university education course is one of the most critical training courses in every person's life. As the period of school education transition, and objective thinking is the entry into the abstract and adolescent thinking that comes with entering the job market and assuming significant responsibilities in life ( 5 ). It should be stated that people studying critical thinking skills at the university are always looking for reasons and evaluating them in real life and resisting misleading prejudices ( 13 ). Therefore, if students engage in critical thinking skills and critical thinking values are developed in them, social benefits will have to be obtained that will be so huge ( 14 - 15 ).

Amini and Fazli Nejad (2000) reviewed the critical thinking skills of the students of the general practitioners of the medical school of Shiraz University of Medical Sciences; the results of this study showed that students were weak in using critical thinking skills ( 16 ).

Mc Grace's study (2003) showed that the mean scores of critical thinking skills of medical students are increasing from year 1 to 4 (except the third year) ( 17 ). The results of Paul's (2014) Delphi study showed that the curriculum should be designed in such a way as to enhance critical thinking and make it possible to evaluate it. Sufficient time needs to be allocated to assess critical thinking ( 18 ).

Agnesno and Marie (2005) in a study criticized barriers to critical thinking, such as lack of faculty knowledge, use of teaching and assessment methods that do not facilitate the critical thinking of the learners, negative attitudes of faculty members towards change and their resistance to change, inappropriate selection process and poor educational backgrounds that do not facilitate the students' critical thinking, insufficient socialization, culture, and inadequacies of education ( 19 ).

Recently, given the dramatic changes in the students' curriculum, especially the volume of courses and the need to integrate medical courses, the attention of the academic education system to curriculum revisions has been criticized. On the other hand, given the integrated complexity of critical thinking, the importance of revising the curriculum becomes more critical. Therefore, the present study aimed to investigate the barriers of critical thinking in the medical school curriculum from the viewpoints of medical education experts. Perhaps, the qualitative recognition of these barriers in the curriculum has made it possible to redefine it to develop critical thinking in medical students and its importance in their future lives and careers.

## Methods

This is a qualitative study aiming to explain the barriers of critical thinking in the medical school curriculum. In this study, using the qualitative research approach, critical thinking barriers in the medical school curriculum were explained from the viewpoint of medical education experts of Shiraz University of Medical Sciences. In this section, semi-structured interviews were used to collect the data. 

The research context was affiliated to Shiraz University of Medical Sciences, School of Medicine, and clinical and basic sciences groups. These environments provided access to qualified teachers. In this study, participants were selected from the Faculty of Medicine in the departments of Basic and Clinical Sciences, and faculty members of the Department of Medical Education and five medical students were selected by purposive sampling method. The sampling was continued until the previous information was repeated, and the content or new nature of the participants were not extracted.

Inclusion criteria were the participants’willingness to participate in the study and express their views, opinions and wealth of information, and teachers with more than five years of teaching experience in the university regarding the concept under the study. 

Before collecting the data, the researcher provided explanations about the goals, process and conditions of the interview. The data were then extracted using semi-structured interviews with open non-led questions and collected using a few guide questions. The interviews were started with general questions such as “What do you mean by critical thinking?”, “Have you experienced critical thinking in the curriculum?” and "How did you incorporate critical thinking into the curriculum?", Or "What are facilitators or inhibitors of critical thinking in medical students' curriculum? Explain it. Interviews continued based on the responses of contributors and with the help of exploratory questions, such as "Explain more. What is your example?, or What do you mean? ". Subsequent questions were based on initial responses of individuals or analysis of previous interviews. The duration of each interview was about 30 to 45 minutes, considering the faculty members' time. Interviews continued until data saturation, and sufficient information was received. After the participants' agreement interview was done in a relaxed and comfortable environment. All interviews were digitally recorded and verbatim word by word. Data analysis was carried out simultaneously with data collection. The simultaneous analysis of the interviews provided the main people with access to further interviews, resulting in more valuable information.

In the present study, to analyze the data obtained from interviews, we used a qualitative content analysis approach in the conventional way ( 20 ). The analytical unit was firstly determined by rewriting the interviews right after each section. Data analysis began by repeated reading of interviews, so that the researcher obtained an overview of the concept. Subsequently, based on the descriptions of the participants, semantic units containing meaningful sentences and words were identified and analyzed. In this way, the order of raw codes was extracted based on the nature of the data.

Continuously, the process of reduction and compression of semantic units was done. Thus, each semantic unit was named and conceptualized in terms of its implications. Then, the same necessary codes were merged and organized and categorized into the first classes.

We tried to group the codes that are more similar to each other in the same categories. In other words, the codes categorized within the classes were homogeneous and heterogeneous with other classes. Then, the primary classes were merged based on the relationship between them, and the main classes were formed. In the final stage, the researcher tried to discover the central themes by comparing and revisiting the classes and subclasses. At this stage, by integrating the same main classes, the content of the barriers to critical thinking was derived from the curriculum of medical students.

Strobert and Carpenter (2011) have proposed four criteria of validity or reliability, transferability, and verifiability for evaluating and validating data in qualitative research that have been used in this research ( 21 ).

### *Trustworthiness*

In the end, the extracted themes were presented to the participants (member check) and it was found that some changes were needed. The themes were also provided by one of the qualified qualitative experts who tried to present all the opinions of the experts in conveying the concepts to the audience (peer check).

## Results

In this part of the study, the findings of the research are presented in order to explain and identify the barriers of critical thinking in the medical school curriculum by content analysis method. In general, the analysis of the interviews in this study was that during the data comparison process, 47 codes were initially extracted from the interviews, which were subdivided into nine main categories after data analysis and integration. Finally, from the clean codes, 14 subcategories, 8 main classes, 2 themes (socio-cultural conditions and traditional and unchanging system of education) were formed. Table 1 shows the themes, categories, and subcategories.

**Table 1:** Subjects, main categories, and subcategories extracted from the study

Themes	Categories	Sub-categories
Socio-cultural conditions	Resistance to critical society	The Linear Thinking-Intellectual Dogmatism
		Obedience in the system
	Intellectual tension	Anxiety, stress, and fatigue
		Curriculum Overload
		Not-Organized Thinking
	Personality characteristics	Lack of confidence
		Lack of motivation
		Curiosity in search of information
Traditional and immutable system	Lack of understanding of society's need for criticism	Lack of attention by curriculum planners to incorporate critical thinking and high-level skills into the curriculum
	The rule of traditional teaching Pattern	Lack of freedom to comment
		Not providing a questioning environment
	Lack of critical thinking skills	Lack of specializing in critical thinking
	Ineffective evaluation	Lack of proper feedback, routine and inappropriate evaluation methods
	The difficulty of critical thinking Training	Unwillingness to participate in critical thinking training

### *A) Socio-cultural conditions*

Providing the right educational and cultural conditions that separate the individual from school education and provide rapid and widespread access to the community and labor market in a college education course is useful in fostering a critical personality. . Given the importance of this topic, social-cultural conditions were the first themes extracted from the qualitative data analysis in this study. The theme consists of three main categories: "resistance to critical society," "tension," and personality factors, which are described below in each of the main categories and subcategories of this theme.

**1. Resistance to the critical community:** Based on the experiences of the study participants, resistance to society and belief in the superiority of collective thinking over independent thinking as the first significant category were the two subcategories of "linear thinking-intellectual dogmatism" and "systemic obedience."

**1-1. The Linear Thinking-Intellectual Dogmatism**

Based on the concept of this subcategory, the participants in this study acknowledged that in the current situation, some linear thinking prevails, according to which, without sufficient knowledge of the community's need for criticism, medical schools only train students in a parrot-like and linear fashion.

One contributor commented on positive support for critical thinking:

*"When we train the learner with traditional methods, like lectures, and do not use collaborative educational methods and class discussions, the focus and accuracy will be reduced, and the quality of education will also be affected."* (Professor-Contributor No. 8).

The same participant said:

*"... there are many students, and it is not easy to control them in the collaborative space and the discussion in the class; on the other hand, the increase in the volume of the course contents and the need to teach all the concepts in the classroom does not provide the opportunity for participatory teaching; therefore, there is not enough opportunity to interact or educate. "* (Professor-Contributor No. 8).

An increase in the volume of the course content, a large amount of documentation in the curriculum, unbalanced workload, and the need to maintain the conditions and get used to implementing traditional training in the classroom are the items participants referred to in expressing their experiences in examining the causes of linear thinking.

**1.2. Obedience in the system**

Submissiveness in the system is the second main category, and this means that obedient thinking is the product of linear thinking. Contributors mentioned the critical role of obedience, stereotyping, and non-positive mental flexibility in the learning system of learners. One of the participants said:

*"The current educational system of the university directs the learner to follow a routine and uniform process and expects them to take a clear and coherent framework. In this atmosphere, the student moves toward a kind of stereotype and cliché. "* (Participant No. 3). 

One of the contributors believed in the students' desire for traditional education and use of simple mental stereotypes rather than using specific solutions and says:

*"Students are interested in temporary and common solutions because of their past education during school time; they are reluctant to participate in class discussions and find creative solutions to the problems."* (Professor- Contributor No. 4).

Another participant believed that:

*"Given that the professor has provided space for collaborative learning, in most cases, mental prejudice and lack of critical thinking of learners into specific ideologies and beliefs and mental stereotypes on a particular issue prevent the use of critical thinking skills and the emergence of creative solutions in the classroom."* (Professor- Participant No. 12). 

**2. Intellectual tension**

Intellectual stress is the second major category of socio-cultural conditions based on the participants' experiences, consisting of three subcategories of "anxiety, stress and fatigue," "curriculum overload," and "mental messiness." 

**2.1. Anxiety, stress, and fatigue**

Anxiety, stress, and fatigue were one of the aspects of distracting factors that triggered specific social conditions and factors such as war and sanctions, and ultimately prevented critical focus and thinking on the issues.

One of the participants stated his experiences on stress and exhaustion of learners in the educational environment:

*"Sometimes in the classroom I find that some students do not have enough focus on lessons because of fatigue, and sometimes they take a nap in the classroom; stress makes them sick, perhaps due to the educational method, which has taken the opportunity of students to participate and think. "* (Professor- Participant No. 1). 

**2.2. Curriculum Overload**

Providing enough space and time to think about the subject and participate in different topics that were experienced by most of the participants can be useful in enhancing critical thinking skills.

In this regard, one of the participants believed that:

*"Due to the compactness of the curriculum of the basic sciences of students, there is no opportunity to present the concepts collaboratively, and they must quickly teach the subject, which removes the atmosphere of thinking and discussing from students."*(Student- Participant No. 3).

Also, another participant believed that:

*"A large amount of course content in the basic sciences causes the dispersion and confusion of students and takes the opportunity to discuss and think about different concepts from the students."* (Student- Participant No. 6).

**2.3. Not-Organized Thinking**

Most students expressed distraction, slackness, and lack of mental focus on various subject areas in their experiences, which reduced or neglected the use of critical thinking skills.

Another participant believed that:

*"Some participants at the time of entering the university do not have any specific plans of their future, and they are also confused, unobtrusive, and wacky in the classroom, and they usually go from branch to branch."* (Professor-Participant No. 5).

The other participant noted that:

*"The confusion and distraction of learners during the education period and the lack of focus on the concepts of learning may prevent the acquisition of new information."* (Student-Participant No. 14).

Another participant in her experience stated that:

*"Many entrance exam counselors advise the learners to endure the hardships of studying for a course that will make them more comfortable and free after passing the exam, which will confuse the learners."* (Student-Participant No. 15).

 Most learners in high-level mental situations often think of routines and familiar affairs, which is caused by the entrance exam thoughts leading to lack of attention to innovative and new options. This is due to brain dispersion ?? and slackness and, on the other hand, to thinking in a particular mental context that prevents a person from thinking and creative solutions.

**3. Personality characteristics**

Faculty members believe that increasing self-confidence is one of the essential needs of all people, especially students. We usually get the most out of those who have high self-esteem because these people have characteristics which make loving people and are often more admired. These people cause classroom dynamics, challenge different issues and opinions, and support their ideas in various topics and provide them with acceptable collective arguments. The existence of such learners is necessary for every educational environment. This theme consists of three categories: "lack of self-esteem," "lack of motivation," and "lack of curiosity in the search for information."

**3.1. Lack of confidence**

Based on the experiences of the participants, one of the characteristics of the learner's personality that is effective in strengthening critical thinking is self-confidence.

One of the participants believed that:

*"Learners who have higher levels of self-esteem in the learning environment and lessons of discussion use critical thinking in decision making and solving different issues, and do not retreat from class positions in class discussions and debates. They have various reasons for their answers, which make a significant contribution to classroom dynamics. "*(Student-Participant No. 12).

In this regard, one other participant also said:

*"One of the significant barriers to developing critical thinking is the very nature of personal thinking processes. Individuals with high self-esteem seek information and exchange their thoughts in the classroom, and this results in classroom dynamics and better education and higher quality for all learners. "* (Professor-Participant No. 4).

The findings suggest that learners with high self-esteem are more successful than their classmates. In other words, learners with a higher level of self-esteem have a better job and a better position than the others and achieve more success in society. This is why increasing self-esteem is one of the essential steps in the success of critical thinking in learners.

**3.2. Lack of motivation**

Participants' experiences confirm that lack of risk-taking, low self-esteem, and lack of motivation to risk are the primary barriers to critical thinking in learners.

One of the participants stated that:

*"Teaching critical thinking is useless to learners who do not have the incentive to use critical thinking in the classroom."* (Professor-Participant No. 3).

The participant adds that:

*"Learners in the classroom have to work together with faculty members and other learners to analyze issues and topics and find the right solutions actively. To achieve this educational success, the learners should have sufficient motivation as a prerequisite. This motivation can be enhanced with various rewards such as class support and encouragement and feedback, and even so, to say, it can motivate learners to become more risk-averse. "*(Professor-Participant No. 4)

**3.3. Curiosity in search of information**

Participants have experienced that curiosity is the key to developing learning intelligence, and it is a personality trait of some people. Having this feature strengthens critical thinking skills in classroom debates and topics.

One of the participants stated that:

*“The faculty members must allocate more time for negotiation and show critical thinking while they are teaching. They should also use methods to raise the students’ curiosity.”* (Professor- Participant No. 9).

Having some personality traits such as self-confidence, curiosity, flexibility, and creativity, willingness to think is useful in developing critical thinking.

### *B) The traditional and unchanging system of education*

According to the participants and their experiences, the traditional and unchanging system is hard to change, and it doesn't accept any innovation. Believing in this method of passive teaching and learning is a common practice in many academic educational settings. In recent years, considering the positive effects of modern teaching and their acceptance in academic settings, the use of modern teaching methods has been considered that can provide a space for the use of critical thinking skills. The five main categories included "lack of understanding of the community needs for criticism," "dominance of the traditional teaching model," "lack of experts in critical thinking," "ineffective evaluation," and "the difficulty of critical thinking education."

**1. Lack of understanding of social needs for criticism**

Based on the experiences of the participants, the role of criticism in the curriculum is unclear, and the need for criticism seems urgent. In this regard, fostering critical thinking and developing an appropriate curriculum by incorporating critical thinking concepts and skills can be very useful.

One participant believed that:

*"The current curriculum concepts are usually incompatible with the needs of learners and today's society, and the need to rethink curricula is very much felt, while students need to educate themselves on critical thinking and not just accumulate much content in their minds."* (Student- Participant No. 14).

Another participant adds that:

*"Teaching students to use critical thinking skills helps them to solve real-life issues and situations in their community and their career prospects, which should be specifically addressed in the curriculum."* (Professor- Participant No. 9).

Another participant believed that:

*"Curriculum design is fundamental considering the space for research, hypothesis and idea development in different topics and appropriate timing for learners to utilize critical skills, and it will also have an impact on the future of students' careers and community needs. "*(Professor- Participant No. 2).

Critical thinking can be improved through needs assessment and curriculum development which includes essential components such as goals, content, teaching, and evaluation process to foster the learners' critical thinking to achieve creativity and academic achievement.

**2. The Rule of Traditional Teaching Pattern**

New ways of planning and integrating student courses based on the participants' experiences have been considered. The use of these new teaching methods provides the appropriate space for practicing and critical thinking skills in learners. However, in practice, traditional teaching patterns usually preclude the comprehensive and complete implementation of new teaching methods.

One participant believed that:

*"Most teachers are interested in using traditional teaching methods, and learners have become accustomed to these methods, and they are more than happy to be provided with written content and only memorize the content. "*(Professor- Participant No. 10).

Another participant added that:

*"Students usually fail to think multidimensional in a variety of subjects around the classroom, and they usually continue one-dimensionally and just memorize the lesson content and avoid participating in different topics or thinking about different subjects."* (Student- Participant No. 16).

This section notes that the use of new teaching methods can provide a space for the use of critical thinking skills.

**3. Lack of critical thinking skills**

Participants in their experiences stated that faculty members did not have sufficient mastery of critical thinking concepts at present. The use of highly qualified teachers in modern teaching methods and the provision of a suitable environment for student learning has made critical and questioning skills essential in the current teaching environment.

One participant added that:

*"Young teachers are usually interested in new teaching methods in their teaching and provide a space for discussion, suggestions, and criticism of free and independent concepts and thinking, which is usually reduced by the work experience of the faculty members, as well as their lack of time. "* (Professor- Participant No. 6).

Another participant believed that:

*"Some subjects are not considered by the teachers because they cannot be taught in traditional teaching methods. These topics will only be transferable to collaborative discussions or classroom discussions with learners."* (Professor- Participant No. 5).

**4. Ineffective evaluation**

The experiences of the participants in this study confirm that evaluation without feedback and taxonomy of critical thinking is ineffective in evaluating the learners, and providing feedback in this space is one of the most difficult challenges to which the teachers are faced. On the one hand, the provided feedback must be truthful. On the other hand, feedback should be given to learners at the right time in order to be active and efficient. Providing students with the right feedback at the right time can be one of the appropriate methods of evaluation that enhances their critical thinking skills.

One participant in providing feedback to the students mentioned that:

*"Despite the fact that students enjoy receiving rewards or correcting their bugs during their teaching, university faculty members, due to their busy work and high volume of teaching materials, avoid providing a collaborative learning environment with questions and answers while teaching and appropriate evaluation and feedback and they usually finish the teaching with traditional teaching methods, which can affect the quality of teaching. "*(Professor- Participant No. 11).

Good feedback always comes with positive things that help the learners feel more at ease and take more steps to succeed, advance and ask questions that require excellent thinking skills to be answered.

**5. The difficulty of teaching critical thinking**

Providing students with critical thinking skills in educational settings is very difficult, making it less likely to use this teaching method in the classroom.

One contributor believed that:

*"Due to the size of the curriculum and the need to train all the resources, teaching traditionally seems more appropriate because taking classes in a new way of teaching will waste the time and not transfer all the subjects to the students."* (Participant No. 6).

Another participant believed that:

*"Holding the classroom in new ways is time-consuming and difficult, and the trainees usually do not make effective use of the available space, so the teaching time is spent on unnecessary discussions instead of transferring lesson concepts that will not do better for students than wasting class time."* (Participant No. 7).

## Discussion

 

It is necessary to develop critical thinking for a learner, due to the increased amount of information, judgment and decision making, and the improvement of the individual and professional life, ( 22 ). Analyzing the data showed that the two themes of cultural-social conditions and the unchanging traditional system are the main barriers of critical thinking in the medical school curriculum.

Cultural-social conditions constituted the first key infrastructure of critical thinking barriers for medical students. The findings of the present study showed that lack of support for independent and critical thinking was one of the most critical issues that most participants mentioned. Rezaei and Haqqani (2015) have studied the causes of resistance to change; they considered the lack of participation of learners in classroom education and values and the shortage of support for creative thinking as one of the most important reasons for resisting changes. What is more, he believes that in order to implement the change successfully, we need to find appropriate solutions to overcome these causes ( 23 ). The results of this study are consistent with those of the present research.

On the other hand, submissiveness in the system is another aspect of this dimension, which is incompatible with the results of the research by Graham (1991) ( 24 ). He has concluded that older people, especially if they are single, look at obedience superficially, and try to solve the issues more creatively and discuss different issues more critically. This result is also consistent with those of the Kalantari and Babayan’s (2014) study ( 25 ).

According to the findings of this study, distortion factors are one of the other themes of this research. Anxiety, stress, and fatigue were the most important aspects of this research. Najafian Zadeh et al. (2014) have concluded that the critical thinking of the students in our country is weak, and one of the causative factors was anxiety and fatigue in students, which was due to the high volume of the courses ( 26 ). Since critical thinking is not considered as an essential dimension in the teaching of students during the educational process by the educational system, and critical thinking is a complex mental process that provides flexibility, proper response, correct predictions and rational decision making of the students in different situations, it is necessary to pay attention to this issue by improving educational patterns, which is consistent with the results of this study.

The lack of enough time to think was another obstacle to this dimension. In this regard, Sharifi et al. (2016) concluded that the use of the assistants of critical thinking capabilities was weak, and this was because they are so busy and do not have enough time during the study that is in the same line with the results of this study ( 27 ).

Intellectual stress is the last theme of this study, the most important aspects of which were anxiety, stress and fatigue, an overload of curriculum, and intellectual fatigue. 

In the research by Durova et al., ( 28 ) participation learning was also a method of developing critical thinking; also, discussing and revealing new ideas and evaluating other people's ideas for developing critical thinking and problem-solving skills and participatory learning were usd. By creating learning management and meaningful experiences and stimulating the learners to think, faculty members have a facilitating role in this issue.

The last subject of this research was the personality factors that included issues such as lack of self-esteem, lack of motivation, and curiosity in seeking information that was attractive to the participants. In this regard, the results showed that, in the viewpoint of the experts participating in this study, one of the most effective barriers to the use of critical thinking is personality barriers. The investigation of numerous texts has shown that having some personality traits such as self-esteem, curiosity, flexibility, creativity, and willingness to think is valid in critical thinking skills ( 13 ). Therefore, the absence of any of these personality traits in individuals can be a barrier to critical thinking. Some scholars believe that critical thinking embraces something beyond the aspects of intelligence and individual performance, and other factors such as emotional and personality traits also affect it ( 14 ). As a result, it seems that before entering the clinical field, it is necessary to go through psychological courses to improve individual characteristics and, thus, increase the skill in critical thinking because the goal of teaching critical thinking is to educate people who are far from personal prejudice and who are careful about their work ( 13 ).

In the personality aspect of the students, "lack of self-esteem" was considered by the experts participating in this study to be the most crucial factor in not using critical thinking, which could cause passive confrontation with events and referral to the authorities. Therefore, along with nursing lessons and in-service training, it is necessary to use strategies for increasing the self-esteem of the nurses and students.

The second theme of this study was the traditional and unchanging system of education which consisted of five categories: "lack of understanding of the need for critical society," "rule of traditional teaching," "lack of experties in critical thinking," "ineffective evaluation" and "difficulty in teaching critical thinking." As noted, the lack of understanding of the community's needs for criticism was one of the main categories of the subject, and it addressed issues such as the lack of attention of curriculum planners to critical thinking and high-level skills in the curriculum. In this regard, researches have been done to confirm the impact of goals on the content, teaching methods, and evaluation as curriculum elements in fostering critical thinking. Based on Vagra’s research (2007) in Lipton School, which teaches in-service teachers, consideration of structure, theory process, this is an essential indicator that critical thinking skills can be achieved through the selection of goals, content, and processes and methods ( 29 ).

Another aspect of this theme was the ineffectiveness of the traditional model of teaching and evaluation. In other words, enhancing motivation to use thinking as the key to improving the quality of students' critical thinking is typically overlooked in the formal curriculum of students; therefore, one of the most effective measures in this regard is to improve the attitude aspects of critical thinking, considering the hidden curriculum function in higher education. In this regard, the researches by Alipour ( 2 ), Sharifi ( 26 ), and Shariatmadari ( 30 ), have neglected development of questioning, analysis, composition and evaluation skills and judgment in learner curriculum, and the lack of attention to the hidden curriculum has been considered one of the significant barriers to developing critical thinking skills. Research by Talebzadeh et al. (2009) has also considered attention to hidden curriculum elements as one of the influential factors in fostering critical thinking ( 29 - 31 ).

Lack of experts in critical thinking was one of the other main categories of this theme. In this regard, Cook and Mull's research points to learning-based approaches to promoting critical thinking, problem-solving, active participation, identification of learning needs, creative discussion, peer learning, and the integration and production of knowledge that make learning realistic, entertaining and attractive.

The difficulty of teaching critical thinking was the last category of this theme; in this regard, research by Drewa et al. found it necessary to discuss and reveal new ideas and evaluate the ideas of others to develop critical thinking and problem-solving skills and collaborative learning, and young teachers have been identified as facilitators or leaders of learning and creators of meaningful experiences that stimulate the learners to think.

According to the results of research done in this dimension, most educational systems tend to have a fixed curriculum content in the curriculum without the use of new, collaborative learning methods in learners, all of which discouraging the students from using critical thinking skills.

### *Application of findings*

The findings of this study provide an overview of the critical thinking situation in the academic environment and the barriers of utilizing critical skills in medical students that can be a practical step in revising and designing curricula with a more comprehensive view of new educational practices, especially concepts and critical thinking skills.

### *Study limitation*

In this study, access to experienced professors in this field was difficult nationwide. However, we sought to use any source of information, scholars, and students in this field.

## Conclusion

The educational system Our educational system needs a development which relegates the education from extreme reliance on old knowledge; brings prospects into thinking, intellection, research, creativity, and innovation;blossoms the students’ talents; deepens the critical spirit of critique and review; boosts the self-esteem and self-confidence; and promotes the educational, biological, technical and professional skills in the young generation. Such a change will be possible by changes in the attitudes of the faculty members, planners and curriculum in formulating strategic plans for goals and content, and also teaching and evaluation methods for curricula and attention to the importance and necessity of developing critical thinking at different levels of education. 

In general, the cultural and social conditions of the traditional and unchanging system of education were two significant barriers to the implementation of the critical thinking program, which seems to overcome these barriers to provide critical thinking concepts and skills in medical students' curricula. Therefore, it can be concluded that the reform of the educational system in educational and clinical environments is one of the strategies for increasing the use of critical thinking.

Academic faculty members can help to improve the critical thinking skills of their learners by creating an atmosphere of inquiry and an appropriate platform for negotiation. Establishing workshops and meetings for familiarizing and enhancing search skills, questioning techniques, and evaluating the value and content of information, as well as holding workshops, can reduce the significant barriers to critical thinking development with effective use of information by students. Therefore, based on the results of this study, it is recommended that an effort should be made to transform the classroom environment into the interface between learners and faculty members through the elimination of the existing barriers.
